# Seeking offsite help: a study of online support access for families of adolescents with depression

**DOI:** 10.3389/fpubh.2025.1463900

**Published:** 2025-02-24

**Authors:** Chen Zhang, Lefan Zhou

**Affiliations:** ^1^College of Liberal Arts, Journalism and Communication, Ocean University of China, Qingdao, Shandong, China; ^2^State Key Laboratory of Media Convergence and Communication, Communication University of China, Beijing, China

**Keywords:** families of adolescents with depression, family communication, WeChat group, online social support, adolescent depression recovery

## Abstract

**Background:**

As the smallest social unit, the family is the primary source of social support for adolescent patients to withstand chronic diseases. Several rehabilitation programs have found that involving family members in the treatment process can result in greater success. However, families struggle to provide adequate support for the recovery of adolescent patients when adolescent depression occurs.

**Methods:**

This study examined the WeChat group for parents of adolescent patients in the “DuGuo” community, and used participatory observation, social network analysis, and extensive interviews to investigate the online support received by families with adolescent depression.

**Results:**

It was found that compared with physical diseases, adolescent depression recovery is a systemic problem, requiring all ecological contexts for adolescent growth to provide relative support. Under structural pressure, families with adolescent patients urgently require diversified social support such as medical consultation, emotional comfort, guidelines to return to school, and life planning. Insufficient offline social support leads parents to seek help from the Internet community.

**Conclusion:**

Widespread factors such as communication constraints and hidden alienation in the nuclear family, render the parent–child relationship an important variable in combating depression. WeChat groups indeed provide a platform for parents with depressed children to seek help, but the real challenge for these parents is how online support from “off-site help” can be used in the family context and positively affect adolescent patients.

## Graphical abstract

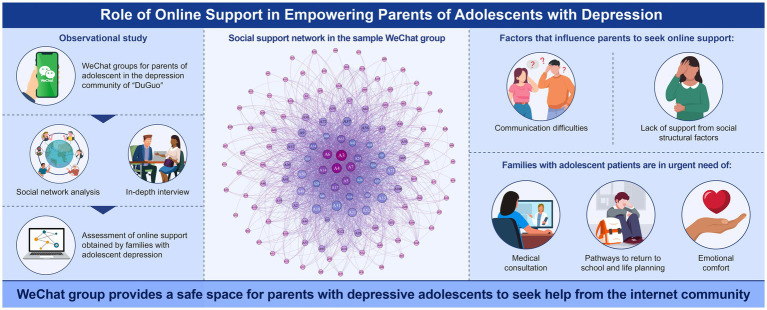

## 1 Introduction

Online social support extends the research scope of social support to cyberspace, whereas the source of social support has gradually changed from “old acquaintances” in offline society to “strangers” in online communities. Social media constantly satisfies the personalized health information demands of various groups, whereas depression groups can establish a health community that fosters mutual support among members through the aggregation function of the Internet. Virtual communities based on social attributes are vital in knowledge exchange, spiritual encouragement, communication, and sharing, and their effectiveness has been tested in numerous empirical studies (1).

Depression is a type of mental illness. The main clinical symptoms include long-term depression and energy loss, which further affect people’s normal cognitive, physical, and behavioral activities (2). Rapid modern civilization increases the strain of individual progress, resulting in depression onset at a younger age. Consequently, the problem of adolescent patients dropping out due to depression and returning to school after recovery causes distress in these families. A report regarding the 2022 Youth Mental Health Survey released by the Institute of Psychology of the Chinese Academy of Sciences, indicated that 14.8% of the teenagers who participated in the survey were at risk of varying degrees of depression. According to the National Depression Blue Book 2022, 86 and 68% of patients believe that the main causes of depression are emotional stress and parent–child relationship, respectively. Notably, 30% of patients with depression are below 18 years and 50% are school students.

In family sociology, the healing capacity of the family is regarded as a crucial factor in disease-causing or recovery. This is because the nuclear family or next-level relatives provide social support to patients (3). Studies based on patient aspects cannot summarize the complexity of the recovery process in depression groups. This study focused on families with an adolescent experiencing depression and defined its concept as families in which juveniles suffer from depression and those in which parents are aware of it and begin seeking help from the Internet community. The majority of parents are willing to help, and actively seek online support on behalf of their children, so that, to a certain extent online support does not directly affect patients.

Families with adolescents suffering from depression, frequently exhibit cross-generation help-seeking behavior, with parents as the core actors. Based on this observation, discussions on adolescent depression recovery should not be limited to the patient level. Family members, especially primary-level relatives, should be included in the analysis scope. Notably, the family as the basic unit should be studied from a holistic perspective.

Moreover, the inability of families to provide support at the social structural level for adolescents with depression is the main reason for seeking online support on social media. An ideal rehabilitation system for adolescent patients should include doctors, families, schools, society, and other factors. The rehabilitation challenges faced by adolescents who drop out of school are not only pressures from school and society, but also the rehabilitation environment within the family. This is mainly due to the lack of effective communication between parents and children, and family communication patterns determine the extent to which parents are able to pass on the help they receive from the online community to their adolescents.

Simultaneously, the adoption and consent of adolescent patients to online support determines how it affects them through the intermediary role of parents and how effective it is. The family communication mode affects their adoption behavior. Therefore, this study focuses on the media use behavior of families with adolescent depression, using their online activities as the investigation subject, and discusses the following questions.

RQ1: Why do the parents of adolescents with depression seek online support? How can parents behalf depressed children to seek online social support when the family’s internal support is insufficient?

RQ2: What are the characteristics of social network support in the families of adolescents with depression? How does the online support received by parents affect adolescents who are not in the WeChat group, and what are the challenges?

## 2 Literature review

### 2.1 Online support practices in the mental illness community

Online health communities have gradually transitioned into a useful tool for patients with mental illness to disseminate psychiatric knowledge, advocate positive health awareness, and exchange social support (4). Social support can alleviate the negative effects of health problems and improve the adaptation level required to revert to normal life (5). Similar experiences of illness and specific demands lead individuals to unite and establish a support forum based on understanding and empathy.

Groups with depression on various platforms have established unique online support scenes: depression Super Topic on Sina Weibo creates a dialog channel for posting and commenting, which is dominated by emotional and instrumental support. The prevalence of online social support encourages groups with depression to transform into “the positively weak” (6). The main role of the Baidu Post Bar in depression is to provide information and emotional support. Members would frequently report positive emotional support when discussing pessimistic and negative coping mechanisms (7). Discussions among users of the North American Depression Forum^1^ focus on information and emotional support, allowing individuals to accumulate sufficient social capital (8). Facebook can provide social support for mothers with postpartum depression, helping them improve parenting skills and reduce depression (9). Patients with mental illness using Twitter to promote mental health shows that social media can support them emotionally and increase their chances of experiencing mental health services (10).

Online social support complements traditional social support in two aspects: expansion of the support context and interaction between the supply and demand of social support. The expansion of the support context provides a support context not limited by time and space; whereas the interaction between the supply and demand of social support provides more interpersonal choices in social support exchanges based on online scenes (11). Due to the restriction of access mechanisms, the social relationship established by the WeChat group enables the Weak Ties between online strangers to transform into strong ties and extends the potential relationship to intensify the reciprocity among group friends.

Notably, the WeChat group is an important carrier for the acquaintance community and a connection channel for strangers to establish relationships, which is a metaphor for the “semi-acquaintance” society with strong and weak relationships (12). For example, the interpretation of WeChat texts of college students with depression shows that WeChat groups can incubate practical strategies to correct excessive medical treatment, alleviate group exclusion, and enhance self-identity (13).

Certainly, social support can sometimes have negative impacts, and it may also be mixed with negative content. Moreover, the resource exchange between those seeking help and those providing help has never achieved a complete balance. Depression can cause serious language barriers, and this negative impact on the readability of social conversations also exists in online health communities, making it difficult for members to accurately express their own thoughts (14). Research has found that heavy users of social media actually experience less emotional support (15). Online information overload is significantly associated with depressive symptoms over time. Online information inevitably lacks the verbal cues present in face-to-face communication, which easily leads to misunderstandings. When social media burnout occurs, excessive and compulsive online socializing can result in increased anxiety and depression (16). For example, the more time adolescents spend on social media, the more likely they are to have depressive symptoms (17). The active members in WeChat groups control the flow and distribution of health resources, and members in marginal positions are less likely to obtain effective social support. Against the backdrop of a high ownership rate of smartphones, the strong willingness and interest in using mental health apps also indicate that treatment opportunities for psychiatric patients are still in extremely short supply (18). Therefore, it is necessary to carefully examine the influence mechanism of social support on families with members suffering from depression and understand the dynamic relationship between social support and depressive symptoms.

### 2.2 Family communication dilemma behind the offside help

WeChat is extremely involved in life scenes, which improves the efficiency of online support access for vulnerable groups. These groups can establish positive psychological tendencies through social media with instrumental and information support (19). In addition, WeChat has a variety of roles such as instant interaction, online consultation, and appointment registration, rendering it a highly practical health medium (20). Online social support has a dialog attribute because users can share their health status, and obtain disease information and related treatment plans on social media (21).

Online social support emphasizes a type of online social relationship networks, in which patients can realize mutual information assistance, empathetic interaction, and material exchange. Individuals in a relatively stable and supportive patient network, can relish the experience of being cared for, relieve disease or life demands, and promote well-being through collective reciprocity. However, when researchers focused on adolescent depression, they found that parents were more likely to seek help on behalf of their children. They usually invest money, time, and effort in helping their children recover.

Existing studies have confirmed that adolescent patients experience many challenges in family communication, in which parents frequently overlook the information regarding seeking help sent by the adolescent and fail to provide timely and necessary support (22). From the family communication perspective, the manner of communicating in families with depressed adolescents directly affects the “landing” of social support obtained by parents from the Internet.

From the interaction perspective, parent–child and marital relationships have an important effect on family communication. The Relational Communication Theory emphasizes that it is necessary to accept differences and focus on communication between family members, to establish a conducive family relationship (22). Estranged parent–child relationships, ineffective parent–child communication, and low-quality parent–child attachment affect the perception and reception of social support for adolescent patients.

According to the Inconsistent Nurturing as Control Theory (INC), “normal” family members frequently hope to control or reduce “abnormal” family members’ health problems, such as depression and drug abuse, using communication strategies (23). Therefore, it is necessary for “abnormal” members to mind the feelings of “normal” members and strive to maintain their relationships, however, this process can lead to irrational use or reinforcement of punishment strategies. The INC theory can be used to discuss the transmission of parental support in the recovery of adolescent patients in families. The supportive behavior of parents seeking help on behalf of their children cannot provide effective social support for the recipient, or reduce the uncertainty of adolescent patients’ coping with illness when parents cannot empathize with the needs of children with depression (24).

Optimal Matching Theory states that social support can be beneficial only if the controllability of events encountered by individuals matches the support provided (25). When parents do not understand the goals of adolescent patients seeking support, or children’s goals are not consistent with parents’ support, the behavior of parents seeking help on behalf of their children will not effectively help adolescent patients in disease management. Therefore, a study of the mechanism of online support for families of adolescents with depression can further understand the dynamic relationship between social support and depression recovery and extensively explore its practical value.

Comprehensive analyses of the family aspect revealed the guiding role of online support in the mutual assistance process. This study applied the theoretical framework of online social support to explore the communication practices of families of adolescents with depression in WeChat groups, and how group interaction provides them with social support outside the family level, to analyze the structure, types, and characteristics of social support networks in WeChat groups, and examine the factors that influence the implementation of such support in the family context.

## 3 Research design

### 3.1 Sample selection

This study examined WeChat groups in the depression community of “DuGuo.” As a folk self-organizing and mutual-aid community with depression, “DuGuo” has some influence. In August 2017, the first WeChat group of “DuGuo” was established, and the founder was a parent of a patient with depression known as “small MM.” With long-term development, “DuGuo” focuses on the treatment of families of adolescents with depression, and the number of users has exceeded 50,000. It has gradually formed a three-in-one online support system for treatment, growth, and companionship.

In this study, the fourth group of the “DuGuo” parents’ class (coded as group A) is viewed as a case sample to conduct investigations. Group A was a positively connected parent-communication group. The majority of the members are parents of adolescents with depression, who constitute the foundation of the community, and children who are not in the WeChat group are approximately between 12 and 18 years old. The main role of this group was to provide online support for the parents of adolescent patients, such as anxiety relief, disease acceptance, and self-repair. During the sample observation, the number of members in the group was stable at approximately 500 (some parents dropped out of the group or newly joined the group), and the community was highly active, with an average of more than 1,000 messages daily.

### 3.2 Research methods

#### 3.2.1 Participatory observation

Researchers observed and participated in the daily activities of WeChat groups to experience real circumstances and accumulate first-hand materials. Since March 2022, researchers have continuously conducted online observations in Group A. During this period, the information in the WeChat group was recorded daily and compiled into memos, such as dialog texts, interactive situations, topic features, and emotional tendencies.

#### 3.2.2 Social network analysis

All the chat records of Group A from July to September 2023 were randomly selected to form a social support network, and a directed, 1-modular matrix. According to statistics, the data volume in group A was 59,703 pieces, involving 137 users. Then Ucinet6 and Gephi0.9.3 software were used to visualize the data.

#### 3.2.3 Extensive interview

All interviews were conducted using semi-structured approaches, WeChat voice calls, or in-person talks. With consent from the interviewees, the interviews were recorded, written, and promptly sorted into word-by-word drafts. According to the situation, some specific interviewees were revisited once or twice, and each interview lasted for at least 30 min, and the relevant contents were all anonymous.

The “Theoretical Saturation principle” was used to determine the final number of interviews in this study. At the beginning of the interviews, the researchers found specific interviewees using open, relational, and differential sampling methods. As the interviews progressed, when the data were sufficient to answer the aforementioned questions and new information was unavailable, the selection of interviewees was stopped.

By September 2023, two adolescent patients (coded as B1-2) and seven parents of patients with depression (coded as C1-7) were finally interviewed (in Table 1).

**Table 1:** Basic information of interviewees.

Code	Membership	Gender	Age	Vocational	education level	Children’s age	Children’s illness time/year
B1	Ordinary member	Male	14	student	Junior high school	–	–
B2	Ordinary member	Female	15	student	Senior high school	–	–
C1	Companion	Female	49	Teacher	Master	16	2
C2	Ordinary member	Male	50	Civil servant	Undergraduate	15	2
C3	Ordinary member	Male	42	Management	Junior college	14	1
C4	Ordinary member	Male	–	–	–	14	1
C5	Ordinary member	Female	55	Peasant	Junior high school	15	1
C6	Ordinary member	Female	46	Freelancer	Junior college	17	2
C7	Ordinary member	Female	41	Secretary	Undergraduate	15	1

The “–” refers to the information that interviewees refuse to provide or do not have.

## 4 Online support characteristics in parent groups

### 4.1 Holistic networks: high-frequency but centralized interactions

According to the social network analysis method, the calculated results show that the density of Group A is 0.122. Since an ideal structure’s network density value should be one, this indicates that families of adolescents with depression have not yet received sufficient social support in the WeChat group. Additionally, the low density reveals inadequate connectivity of the overall community, because the majority of the parents in the group are concerned with the conversations they initiate and participate in. Thus the communication in this group has a high-frequency but centralized characteristic. This apparent centralization trend shows that many members were isolated because of inadequate online support.

**Figure 1:**
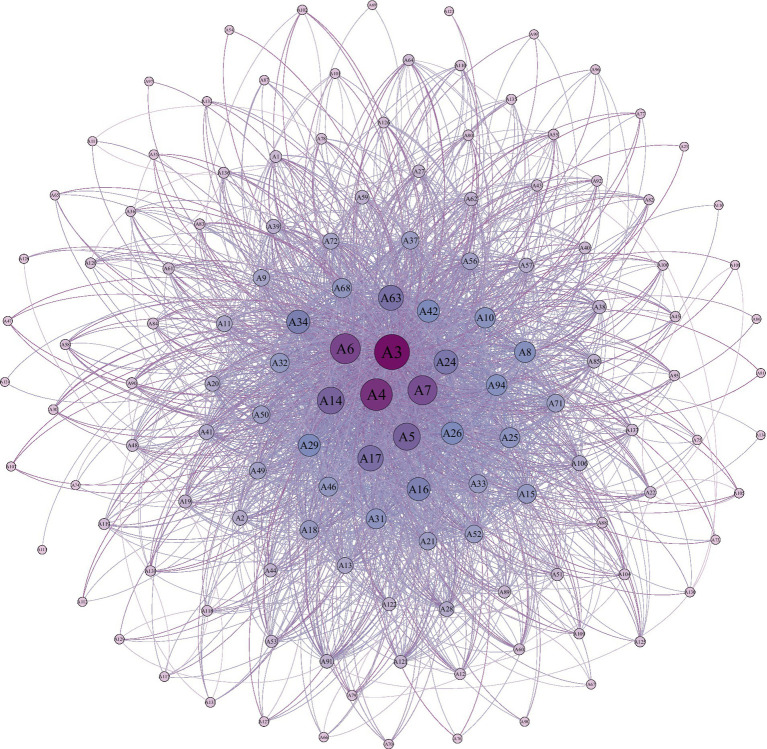
Group A social support network.

In this online dialog of parents seeking help on behalf of their children, spectator parents who are ordinary community nodes experience challenges in receiving personalized help, because they are not active in the community. Furthermore, due to the influence of family communication patterns, it is difficult to effectively transmit online support to their children. As shown in Figure 1, the support network in the WeChat sample group shows a comprehensive radial structure.

Group A support network had 137 nodes and 2,278 relationship lines, implying that 137 members participated in this social support relationship network, and generated 2,278 interactions with one another between July to September 2023. The entire network has a radial shape, gradually expanding from the center to the edges, with connectivity changing from tight to wobbly. This demonstrates two characteristics. First, the central members, such as A3, A4, and A6, link up with other members scattered at the edge of the network, forming an entire support network. Second, members at the center exchange information more frequently, whereas those far from the center increasingly tend to receive support rather than provide it.

When combined with the observation of conversations in Group A, high-frequency but centralized contacts usually occur rapidly and are dominated by regular members. These contacts are rarely chit-chats, but rather communications regarding a specific situation. Social relationships were established in this question-and-answer diagnostic conversation. It is similar to the “personal community” proposed by Claude Fischer and Robert Jackson. Relationships in families are established based on emotions that develop from the family members’ shared experiences. In private life, the emotional relationship developed through the common interaction is the basis for people to support their friendships. Communication in community life is “universalist” (26), therefore, community relationships are essentially exchange relationships, which appeal to the attitudes of respect rather than the feelings toward community members.

### 4.2 Individual networks: silent and crowded onlooking

Cohesive subgroups can reveal whether reciprocal or potential reciprocal relationships exist among the actors (27). To comprehensively determine the composition of the support network of families of adolescents with depression, N-faction was used to explore whether explicit “cliques” existed in the WeChat group.

Two hundred and eighty-five cohesive subgroups existed, with 95 members in the largest subgroup’s online support network. It is convenient to find that the scale of the subgroups in this network is a relatively huge number of recurring members (A3, A4, A6, A34, etc.), exist in more than 280 factions, in which these members usually provide cross-group support. Members, such as A23, A81, A114, and A115 had one or zero factions, and their frequency of communication was too low to achieve cross-group support, indicating that some ordinary members in WeChat groups are still unable to receive adequate social support. Therefore, reciprocity of support fails to cover all participants.

**Figure 2:**
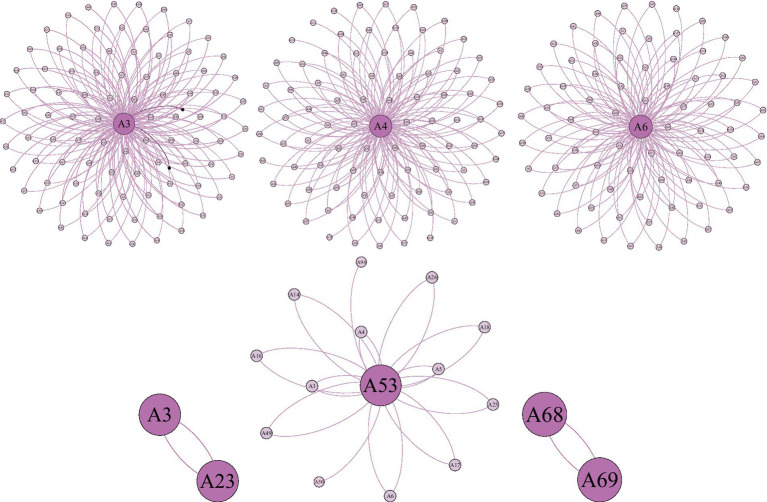
Group A member personal center network (Excerpt).

To further analyze the core-edge structure of the support network, researchers sampled the top three members A3, A4, and A6 with high cohesion coefficients, and A23, A53, and A69 with cohesion coefficients of zero, and plotted their personal center networks, as shown in Figure 2. Members A3, A4, and A6 have high structural similarity, and their personal networks are still centered on themselves with outward radial dissemination. However, the personal networks of A23, A53, and A69 were significantly different. Except for a sharp decrease in the number of nodes and edge relations, the network shape is simple and irregular. Generally, the network has a significant core-edge relationship.

The edge of Group A’s support network is crowded, with more than half of the parents on the periphery of the support activities, thereby re-segregating the disadvantaged group and rendering them more “marginalized.” Previous studies have been positive in discussions on online social support for depression. Furthermore, it is nearly impossible to regard their dual marginalization online and offline because their idealistic conclusions have optimistically estimated this group’s acceptance in mainstream culture. However, the presence of multiple levels of differentiation and differences should not be overlooked.

### 4.3 Reciprocity dilemma of network support

Closeness Centrality refers to the independence of a member of a social network. The smaller the numerical value, the stronger the members’ independence in the process of information exchange, and the less likely they are to be constrained by others’ opinions. The sum of in-closeness and out-closeness centrality can reflect the interaction between the following and being followed. The higher the numerical value, the faster the information flows whereas, the higher the degree of social support, the less likely it is to be controlled by other nodes in supportive communication.

According to Robert Putnam, networks contain mutual obligations to each other, and when individuals realize the importance of social capital, they invest considerable time and effort in foresight to develop or acquire it (27). A social network constituted by Group A implies an expectation of social capital reciprocity, which is generalized reciprocity. This reciprocity among members does not occur at two fixed points, but rather collective reciprocity based on a sense of belonging.

The top 10 members in Group A in terms of closeness centrality are shown in Table 2, including A3, A4, A6, A7, A5, A14, A17, A63, A24, and A34 members. The results showed that the majority in this network had a large gap between in-closeness and out-closeness centrality. Moreover, in-closeness centrality is significantly lower than out-closeness centrality, indicating that the members of the group have a weak capacity to transmit social support to others. The more active members are, the more they can connect with others and access necessary health resources. However, after receiving support from the community, these parents failed to disseminate the support they received.

**Table 2:** Incloseness centrality, Outcloseness centrality.

Ranking	Code	In closeness centrality	Out closeness centrality
1	A3	721.000	995.000
2	A4	732.000	1003.000
3	A6	738.000	1009.000
4	A7	741.000	1012.000
5	A5	749.000	1019.000
6	A14	749.000	1020.000
7	A17	754.000	1025.000
8	A63	756.000	1027.000
9	A24	760.000	1030.000
10	A34	761.000	1032.000

The principle of generalized reciprocity suggests that the costs that individuals incur to establish social connections in a community will not necessarily be reimbursed, but such payments will establish a form of “Bonding Social Capital,” that strengthens the identity and solidarity among community members (28). Consequently, Group A has not been able to form broad reciprocity and this point-to-point single-line interaction has not been able to disseminate community identity to all members.

## 5 Offsite landing of the support within WeChat groups

### 5.1 Support-obtaining: entering motivation of the anxiety community

The main purpose of parents of adolescent patients joining the WeChat group was to promote the recovery of their children from depression and thus transform to a normal life and academic track. In China, the family is the basic unit of social life. Most studies have accepted the premise that adolescents are the subjects of anxiety by default, without realizing that students and their parents are the “anxiety community.” The behavior of parents seeking offside help in cyberspace shows that their families have difficulty coping with the problems caused by their children’s illnesses, such as medication, suspension, and resumption of schooling. This reflects the importance of social structural elements in the recovery of adolescents with depression after a family communication failure.

*If you suspend school, it must be a year. I heard that the resumption process was challenging. She (the child) said that she did not attend the first grade in junior high school again. (A5)*

*The teacher called and severely criticized me for half an hour, and said that the child is a minor, and why the parents have no concern considering all kinds of reasons for the child’s daily absence, asking the teacher, so whether it is allowed to leave or not. The teacher advised parents to be decisive, thinking to transfer their child to a private high school, either suspending, or dropping out, and it should not be the parent’s attitude to help the child pretend to be confused, and procrastinate daily. (A38)*

Similar family communication patterns lead parents to reflect on what they experienced. Thus, by publicizing their previous experiences in the group, they can help other parents withstand anxiety, and the supportive comments popular in the group can help parents receive practical guidance and settle psychologically.

*I think using the words “pretends to be sick” may not be proper. The child was indeed uncomfortable and we needed understand him. He wants to relax at home, relieve their stress, and emotions. You do not know the time he played? I think you were right not to control him (the other issues regarding what time he was playing are concerns, but they are controlling). You should be glad that your child has a mild emotional disorder. When my child had a mild emotional disorder then, because of my ignorance and fear, he deteriorated into a severe emotional disorder, was unable to go to school, and required medical attention. I hope that you can stop the damage over time. (A23)*

Although the process of obtaining social support through online communities cannot be accomplished overnight for sick children, and such spontaneous mutual support activities may not be able to help their children recover instantly, they provide effective emotional support for parents. Some parents seek emotional relief from their parent groups.

*I do not have much energy. I have to work. The child is depressed, and I have to take my husband into account. I need to be cared for. We are a remarried family. I want him to be stable, but he will not coax or comfort me. I have to adjust my anxiety. I would report to the group during anxious and unbearable period. I rarely discuss my child with him. I used to discuss it with him from my heart, but no adequate response, probably because the child was not his. He increased my anxiety. As soon as I discussed the child, he would accuse me of misleading the child, asking me to seek coercive means, such as confiscating the smartphone and computer, and not allowing the child to play. When the child in school called to come home, he did not want me to pick up the child. (A32)*

*I accompanied my son (at the mall) and encountered an acquaintance. Although he did not ask regarding the child, based on his expressions, I thought he knew the child. I wanted to walk in a vacuum. When I return to my hometown and build a villa, let us go there and live together in the future. I do not want to meet acquaintances, but I am not afraid of meeting you guys. (A3)*

To relieve the mental stress of parents, when the “resumption plan” to return to school fails again, the phrase “in good health” is increasingly popular in the group, and “living in good health” has become the “second best” request for parents to accept their children to live with illnesses.

*However, I think my child has no chance in this school. I suggest that she should not go to an expensive high school, lower her requirements, or find a vocational school. The most important thing now is to be healthy, and I do not mind other things, so she can just muddle for three or five years at school. (A36)*

### 5.2 Support-adopting: constraints of family communication mode

Effective social support for adolescent depression has two mechanisms: the “Main Effect” is more direct; whereas the “Stress Buffering Effect” is relatively indirect, which emphasizes the buffering effect of social support on stress compared to the “Main Effect.” Under schooling demands and other educational anxieties, effective social support can help adolescents withstand external demands. Some studies have found that parental support has the greatest impact on adolescent depression in early adolescence (29).

However, from a social structure perspective, it is difficult to provide adequate social support for the depressed because families are the main basic unit of the social system. After adolescents suffer from the disease, their mode of family communication experiences various challenges. Many juvenilesin adolescence and their low conversational communication mode can render family internal communication ineffective. In such situations, parents are incapacitated, and restoring benign communication with their sick children is the main motivation for them to join the group for help.

*In what state does a child need advice from adults? I have never thought of advice or not. My child hates me and says that psychology has corrupted my mind. (A4)*

*I think I am unemotional to provide her with emotional support and pretend that I do not know what to do when I am accompanying her. (A85)*

The Stress-buffering Models suggest that when an individual is stressed, emotional support from family members and “vital others” can serve as a relatively stable internal psychological resource to buffer stress. Emotional support includes actual and perceived support (30). For adolescents, family communication mode during adolescence has a significant impact on how they interpret parental support.

Based on the recipient’s perspective, the types of social support are subdivided into two categories: “Perceived” and “Received Social Support” (31, 32). Parents actively learn and obtain the practices of recovery from depression, which ultimately requires the adolescent patients’ consent. Notably, the effectiveness of the transmitter center was challenged. The effectiveness of the advice needs to be rated in terms of the recipients’ aspects, that is, whether such online support is positively accepted by the adolescents, beneficial to the adolescents, adopted by the adolescents, and affects the relationship between adolescent patients and family members.

The ultimate goal of restoring physical and mental health for parents whose children have been suspended from school due to depression is to return to school and realize a resumption plan. The ultimate realization of the resumption plan depends on the physical and mental capacities of the adolescent patients. A major challenge for parents whose children have been suspended from school is how to connect parents in the group with their children outside the group and match the goals and behaviors of parents and children.

A previous study found that adolescent patients and their parents usually join different WeChat groups. To some extent, this group segregation can help these two groups establish their social networks, however, behind this phenomenon is a type of Internet tracking that reflects the difficulty for family members to communicate with each other offline.

*My daughter is a senior in high school and has to take a day off almost every week, either for a headache, stomachache, or sore throat …… to sleep in the rented room and play games. I told her it was all right to take a day off when she was tired. We usually play separately. I cook, read, and go to physical therapy, and we do not communicate much during the day. I asked her at dinner before: You come home from school so tired every day, is there too much constraints? She answered that it was a stupid school …… I want to talk to her, but I do not know how to do so. I am afraid of mentioning sensitive issues. I thought, it is quite reasonable not to interfere with each other, but I am not sure if I was doing the right. (A55)*

Issues such as low self-esteem, dependence, a lower sense of control, and the demand to be supported under the principle of reciprocity, may persist (33). Resolving such issues needs effective communication on health info between both sides. Also, it should be consciously supplemented by other social support like emotional and respectful support (34). It’s indicated that whether intimate behaviors help establish and maintain a relationship does not rely on the behaviors themselves, but on whether both sides share the same interpretations and meanings of these expressions (35). From the interactive essence of communication, the subjectivity of the social support recipient is increasingly evident in the effectiveness of support, but the network support that flows in Group A ultimately requires acceptance by the adolescent patients outside the group before its effective implementation.

### 5.3 Support-landing: logic behind withdrawing from the group after returning to school

“The family systems theory” considers the family as a basic emotional unit and emphasizes its systemic attributes, in which the emotional and psychological problems of each family member can be addressed (36). In a close-knit original family, adolescents’ illness usually has an apparent impact on parental mental health, invisibly changing the manner in which couples get along with each other, their emotional expression, and relational interactions. Negative emotions are in a vicious cycle in the family, leading to a decline in family roles. Salvador Minuchin, a family healer, believes that the interactive mode in the family is considerably important than the composition of family members.

Family is a social unit, but it is inseparable from the larger systemic ecology of schools, hospitals, and society for the recovery of adolescents with depression. Online support from the community depends on the combined effect of social capital within and outside the family to reach the family context. Internal family social capital refers to the relationship between parents and children, which is characterized by productive parent–child relationships, commitment, reciprocity, and trust. Social capital outside the family refers to the social relationships between parents and other members in the community as well as the relationship between parents and social institutions. James Coleman believes that social capital in the family is the primary approach that the family’s economic resources and parents’ labor capital are transformed into the children’s labor capital (cognitive ability, academic achievement, etc.).

Online support, as a type of “Sunny Day Technology,” optimizes the acquisition of social support for the family, but the optimization function is affected by the existing family communication mode. That is, the convenience and efficiency caused by technology are frequently difficult to realize immediately when the family is in trouble or crisis (24). How the Internet support can reach the family is an important challenge to test the family communication mode. Support from the online community should help their children. An important indicator to test whether the online support has effectively reached the family is whether the student can successfully return to school. Being able to successfully return to school means that the family has overcome this barrier.

*I mean that parents cannot avoid the expectations of returning to school. Indeed it is a challenge to let go. A child in their teens, engaging in misconduct for a day, is a challenge for parents and children. It is good for your family to get out and travel. (A14)*

Through long-term observation of Group A, researchers found that the number of people in the group frequently varies from 495 to 500, implying that withdrawal behavior is not rare in the WeChat group.

Parents who perceived that they could acquire little social support in the group would withdraw from the group silently, whereas a small number of parents would formally say goodbye to all members before leaving the group.

For example, a parent sent a blessing as he left the group:

*You cannot rush to solve your children’s problems or ignore them. I have been joining many groups in recent years, in which some children have kept laying for eight or 10 years. I know a girl in our local area who has suffered from depression for more than 10 years. I talked to her mum, and it is absolutely true. She is now 25 years old and still cannot work, so she applied for low-income insurance from the neighborhood committee. Therefore, it is a warning that we parents should focus on our children’s problems. Many parents in the group are lucky to have their children still in school, and you should cherish it. I will withdraw from the group. I wish all parents and children a happy life and courageously move forward.*

Such public statements of withdrawal raise concerns and questions from other parents.

*What is happening here? Deleted me too. Hopefully the kid improving. (A17)*

Parents in the group who know the conditions of the withdrawing parent respond on their behalf:

*She withdrew from the group, and Mrs. Lee said that her child had returned to school. (A94)*

Some parents whose children have successfully returned to school leave the group, decamping the past collective, and moving on with their children. Some, remain in the group and continue advising others after their children return to school.

In addition, parents demonstrated their daily negotiations with the school in the group, and expected to obtain other parents’ practical experiences and targeted guidance for their children returning to school. This type of interaction established the desire to return to school in the group. Many consultation on returning to school were initiated by new parents in the group. By giving a detailed account of their children’s current condition and reasons for suspension, they hoped to successfully return to school through effective strategies of their groupmates. By learning and self-awareness in the group, some parents adjusted their family communication modes, and online support in the group reached adolescent patients through their parents. The logic of these parents leaving the group after their children successfully returned to school is that they have already gained useful and effective social support, which proves that online support has been transmitted effectively.

By idolizing some adolescent patients who have successfully returned to school and continued to higher education, the group instills a value that “there is no alternative but to resume one’s study on campus” to other parents of adolescent patients. In this community context, parents focus on whether their children can succeed in returning to school, but reduce their vigilance regarding the mental health disabilities of their children. Parents hope that teachers will be lenient toward their children’s refusal to take exams, failure to complete homework, and frequent absences, so that they can complete the minimum level of returning to school, that is, “as long as the child can sit in the classroom, it is fine.” Parents perceive successful returning to school as an important goal for their sick children’s recovery, and are eagerly seeking related information and experience.

*Children suspend school because of depression, how to face, accompany, help them recover from the confusion, whether they can return to school successfully after a year of suspension, and so on …… Many specific problems exist in life. It has been almost two years since my child suspended school in his first year of high school. A slow improvement process was observed with a spiral upward. Compared with the article regarding the five processes of suspension and resumption of school, the general guidelines are consistent. The child did not heal fully. If there is internal motivation, the child returns to school conveniently. (C6)*

It is difficult for the schools and teachers to withstand the consequences that may be caused by adolescents attending school with illnesses. Persuading these students to suspend or transfer to other schools, long-term absence, changes in local education policies, and the retention period limitation of student status have increased the difficulty for adolescent patients in applying to return to school.

Interviews showed that whether hospitals could provide valid recovery certificates during the process of returning to school was another problem for parents, compelling them to consider other measures to avoid medical materials that might reveal their children’s illnesses. Member A8’s child had withdrawn from school, and the mother, cited trauma rather than mental illness as the reason for the withdrawal to issue a certificate of returning to school in the future: “When I went to the hospital to issue a certificate, the nurse told me that it was allowed to provide a certificate but it would have an impact on the child in the future, therefore, the certificate of returning to school could not be issued, so I was asked to issue a trauma certificate. It would be convenient to issue a recovery certificate when the child return to school.” As result some parents believe that the conversations and communication in the group are not beneficial to them; therefore, they avoid the messages or withdraw from the group.

## 6 Conclusion and discussion

### 6.1 Motivations and influencing factors for families of adolescents with depression to seek help online

Looking for assistance, some families of adolescents with depression to seek help from the internet. Mutual assistance, reciprocity, and emotional support in online communities continuously attract members to seek organizations, which effectively promotes offline support through online interpersonal interaction. The study found that structural factors such as educational demands, medical resources, and return-to-school anxiety were major factors in the recovery of adolescents with depression and reasons for parents of adolescents with depression to seek online support.

### 6.2 Structural characteristics and potential risks of online social support networks

From a holistic perspective, the social support network in the group has a radial structure, and the chat-based support interaction in the network frequently fluctuates, with the characteristics of active but centralized dialog. From the relationship perspective, the edge of the network is extremely crowded, indicating that many members do not conduct substantive dialog in the group, but rather randomly capture the information in the group by looking, which potentially hides the risk of re-segmentation. At the individual level, different members of the network behave differently, reflecting the influence of individual factors on help-seeking behavior and their effect when adolescents’ parents seek online support.

WeChat groups have the value of healthy empowerment, and potential to exacerbate the imbalance in social support. Frequent online relationships may reduce willingness to engage in offline activities. For example, the restriction or deprivation of offline support activities in WeChat group can create a transparent “wall” that isolates participants from the real world. The desire for offline communication has becomes increasingly indifferent, leading to blind support worship in the group.

Parents are obsessed with repairing the “broken” family relationship, while struggling with their sick children’s return to school. This dispersed support tendency in the group has overlooked mainstream social prejudice against mental illnesses. “Family support failure” is regarded as the main or decisive factor in the pathogenesis of depression, and has become the main issue in the discussion of family communication modes in the group. Such developments obscure the “contribution” of social structural factors to the high incidence of adolescent depression, which is not conducive for families of adolescents with depression to effectively access social support.

### 6.3 Research limitations and future prospects

This study has limitations. Due to the special identity of the interviewees, the quantity of samples collected is limited. The relation to the privacy of teenagers was not presented in the study. The initial intention of creating and using WeChat groups is to help vulnerable groups gain support, but overemphasizing the role of social media can conceal health equality issues implied by mental illness. Future research should consider the social resource distribution structure, as well as the negative effects of commercial factors on the folk antidepressant community, and focus on the broader social context.

## Data Availability

The original contributions presented in the study are included in the article/supplementary material, further inquiries can be directed to the corresponding author.

## Footnotes

^1^ www.depressionforums.org
